# Community Exercise Program Participation and Mental Well-Being in the U.S. Texas–Mexico Border Region

**DOI:** 10.3390/healthcare11222946

**Published:** 2023-11-11

**Authors:** Alma G. Ochoa Del-Toro, Lisa A. Mitchell-Bennett, Michael Machiorlatti, Candace A. Robledo, Amanda C. Davé, Rebecca N. Lozoya, Belinda M. Reininger

**Affiliations:** 1 Division of Health Promotion & Behavioral Sciences, Brownsville Regional Campus, School of Public Health, University of Texas Health Science Center, Brownsville, TX 78250, USAlisa.mitchell-bennett@uth.tmc.edu (L.A.M.-B.); amanda.c.dave@uth.tmc.edu (A.C.D.); rebecca.n.lozoya@uth.tmc.edu (R.N.L.); 2 Department of Population Health and Biostatistics, University of Texas Rio Grande Valley School of Medicine, Harlingen, TX 78550, USA; michael.machiorlatti@utrgv.edu (M.M.); candace.robledo@utrgv.edu (C.A.R.)

**Keywords:** US–Mexico border, physical exercise, mental health, public health, health behavior promotion

## Abstract

Hispanics are disproportionately affected by low rates of physical activity and high rates of chronic diseases. Hispanics generally and Mexican Americans specifically are underrepresented in research on physical activity and its impact on mental well-being. Some community-based interventions have been effective in increasing physical activity among Hispanics. This study examined data from a sample of low-income Hispanic participants in free community exercise classes to characterize the association between self-reported frequency of exercise class attendance, intensity of physical activity, and participant well-being. As part of two cross-sectional samples recruited from a stratified random sample of community exercise classes, 302 participants completed a questionnaire consisting of a modified version of the Godin-Shephard Leisure-Time Exercise Questionnaire (LTEQ) and the Mental Health Continuum Short Form (MHC-SF). Adjusted logistic regression analyses indicated that those who achieve mild, moderate, and strenuous self-reported physical activity have 130% higher odds (*p* = 0.0422) of positive mental well-being after adjustment for age, frequency of attendance, and self-reported health. This study provides evidence that the intensity of physical activity is associated with flourishing mental well-being among Hispanic adults. The association between physical activity and mental well-being is more pronounced when considering participants engaged in mild levels of physical activity. The study further provides insight into the planning and development of community-based physical activity programming tailored to low-income populations.

## 1. Introduction

Many studies have shown that regular, moderate, and strenuous physical activity prevents the risk of noncommunicable diseases including obesity, cardiovascular disease, and diabetes [1,2]. Hispanics in the U.S. are less physically active across all subgroups than non-Hispanic whites, with socioeconomic factors partially contributing to the disparity [3,4]. A study comparing Hispanic respondents from the 2009 national Behavioral Risk Factor Surveillance System (BRFSS) to the Cameron County Hispanic Cohort (CCHC), a randomly-ascertained cohort of around 5000 Mexican American living in communities near the Texas–Mexico border, revealed significant health disparities in preventive health behaviors, including physical activity. National BRFSS respondents were significantly more likely than local CCHC participants to meet recommended physical activity guidelines (44.14% vs. 33.3%) [5].

Hispanics generally and Mexican American populations specifically are underrepresented in research on physical activity, but literature exists to guide the development of community interventions prioritizing these groups. Interventions focused on addressing barriers to physical activity (e.g., cost, transportation, cultural fit) have been shown to increase the adoption and maintenance of regular leisure-time physical activity among underserved populations, including Mexican American and lower-income individuals living in the Rio Grande Valley [6,7,8].

In addition, community-based interventions have been shown to be successful in increasing physical activity among Mexican Americans by culturally tailoring interventions and increasing social support [9,10]. Social support is associated with regular exercise in all adults, regardless of ethnicity [11,12], and may explain why greater participation is reported for group exercise [13,14,15]. The meaning of social support in the context of its association with increased exercise is multidimensional yet broadly defined as resources provided by other persons, including emotional support and practical support such as encouraging completion of a goal [11]. Evidence also suggests that participation in group park runs was statistically associated with higher levels of participation, satisfaction with exercise activity, and group cohesion [16].

Another area of physical activity research where Hispanics are underrepresented is the impact of physical activity on mental well-being. One study showed an association between physical activity and positive emotions in older Hispanic adults [17]. Additionally, the study showed a statistically significant association between the lack of physical activity and depressive mood symptoms, specifically among these Spanish-speaking participants in the U.S. Texas–Mexico Border region [17]. These findings support the beneficial nature that physical activity, particularly moderate and vigorous physical activity, has an association with positive mental health outcomes. Studies involving the general population also show that the lack of physical activity can have a negative impact mental health, resulting in an increased risk of depression and anxiety, and lower levels of positive mental health [18,19,20]. Very few studies have examined all intensity levels of physical activity and its association with mental well-being. Some of these studies found that light/mild physical activity was associated with positive mental health outcomes [21,22,23], but these studies are rarely with Hispanic populations [24].

In general, all-intensity-level physical activity and its associations with mental well-being have not been evaluated among Mexican American populations, specifically those from low-income backgrounds. One study examined poverty, physical activity, and mental health and found that low-income adults with low levels of physical activity experience poorer mental health outcomes using the Patient Health Questionnaire for Depression and Anxiety (PHQ-4) and Perceived Stress Scale (PSS) [25]. The gap in the literature regarding the relationship between the amount of time performing physical activity, the intensity of that activity, and mental health among low-income Mexican American populations is important to understand given the overall small percentage of Hispanic populations who meet physical activity guidelines. The aim of this study is to fill that gap from a sample of low-income Hispanics who participate in free community exercise classes along the U.S. Texas–Mexico Border to characterize the association between self-reported frequency of exercise class attendance and intensity of physical activity with participant well-being measured by self-report. We hypothesized that not only will moderate and strenuous physical activity be associated with positive mental well-being, but also mild physical activity will provide an additional association with positive mental well-being.

## 2. Materials and Methods

The Tu Salud ¡Si Cuenta! (TSSC) program was developed to address health disparities in chronic disease disproportionately affecting residents of Cameron and Hidalgo counties, located in the U.S. Texas–Mexico Border region. Approximately 1.2 million people, predominantly Mexican American residents, live in Cameron and Hidalgo counties [26]. The poverty rates in Cameron (24.6%) and Hidalgo (28.8%) are more than double the national average (11.5%) [27]. Rates of chronic disease and related mortality are also much higher than the state and national averages [27,28]. Historically, the high poverty rates, lack of access to healthcare, and lack of environments, systems, and policies that promote healthy living result in nearly two-thirds of the population being overweight or obese [5,29]. One-third of adults live with diabetes, exceeding the rate of U.S. Mexican Americans and non-Hispanic white populations [28]. The TSSC program is based on evidence provided through recommendations from The Guide to Community Preventive Services. The guide recommends community-wide campaigns as large-scale initiatives delivering messages using mass media and providing individually focused efforts such as physical activity counseling, health risk screenings, and education [30].

The TSSC community-wide campaign was designed to incorporate mass media, social support, risk factor screening, nutrition and physical activity education, and the promotion of environmental infrastructure and policy change; it is implemented by community partners, and has grown over the last 15 years to include many communities throughout the U.S. Texas–Mexico Border region. As one component of the program, TSSC offers hundreds of free, weekly exercise classes across the region with the goal of increasing individuals’ physical activity minutes per week [6]. Community health workers (CHWs) and certified instructors provide free, daily physical activity and nutrition classes at local parks, schools, churches, and other easily accessible venues in their communities. A variety of physical activity classes are offered in English and Spanish targeting mostly adults and seniors, with a duration of 30 to 60 min. Classes are also offered at mild, moderate, and vigorous intensities throughout the day at times convenient to both working and non-working participants. In addition to nutrition information presented in TSSC monthly newsletters and addressed during physical activity classes, nutrition education programs are offered to complement the physical activity classes including The Happy Kitchen/La Cocina Alegre^®^ cooking classes, Group Lifestyle Balance™ Diabetes Prevention Program (DPP), and the Centers for Disease Control & Prevention (CDC) PreventT2 DPP curriculum. CHWs provide risk factor screening and motivational interviewing to interested participants with elevated blood pressure and/or body mass index to help them identify and work towards personal health goals. Previous studies of the TSSC program have shown that tying physical activity and nutrition classes together, in addition to CHW exposure, leads to positive behavior changes [31].

The TSSC program helps to address physical activity disparities and reinforce positive behaviors by tailoring classes to address the cultural and socio-economic barriers to exercising [5]. Cultural tailoring included the use of local appealing music, recruitment of Hispanic instructors, provision of classes in Spanish, and opportunities for exercise incorporating culturally familiar dance styles (e.g., huapango).

### 2.1. Procedure

Two cross-sectional samples of participants attending community exercise classes between October 2018 and November 2019 were recruited from randomly selected exercise classes. Exercise classes were selected from the total number of classes available during the study period by year. A stratified random sample was conducted to ensure a proportional response from different class groupings. Classes are offered throughout the year but may vary based on venue and instructor availability along with participant interest. Random selection was completed each year to account for this variability. Tu Salud ¡Si Cuenta! offers up to 250 free exercise classes each week at different times and venues across the county in 11 municipalities. A listing of the classes was enumerated by the city for the random selection process. If a city had only one type of class or one class, it was grouped with the nearest city geographically. Classes were listed in numerical order and assigned a class identification number. Using a random sequence generator without duplicates, classes were randomly selected for inclusion in the study. Between 30–40 classes were selected each year in order to provide a 15–20% sample of the total number of classes offered. The number of classes randomly selected in each city was determined by dividing 30 by the total number of blocks. The first sample selection was assessed for proportional representation of all class types being offered at that point in time. Both years, the sample was expanded to include an additional 2–3 classes to help ensure that the sample list captured all types of classes being offered. A total of eight of the 63 selected classes had no class participants or were canceled in 2018 and 2019.

### 2.2. Sample

**Figure 1:**
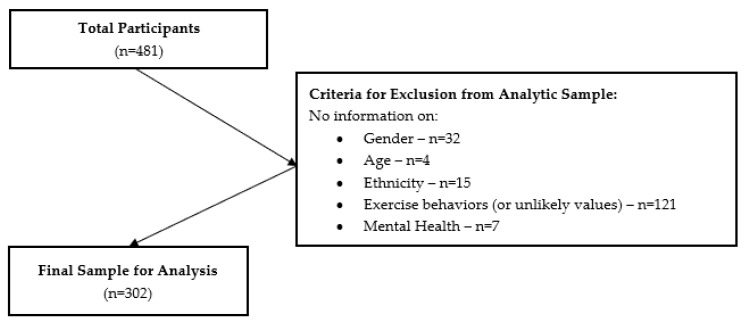
Flow diagram—final sample size.

A total of 481 participants from the 55 randomly selected exercise classes completed study questionnaires in 2018 and 2019. Of the 481 participants who completed the surveys, 302 participants were included in the analysis after excluding those who were not Hispanic, or had missing or incomplete demographic, physical activity, or mental well-being information (Figure 1).

All adults over the age of 18 years in every randomly selected class were asked to complete the questionnaire. The questionnaire was self-administered, took approximately 20 to 30 min to complete, and was available in both English and Spanish. The questionnaire was distributed at the randomly selected classes between October and November the year the sample was drawn, and it was only administered once per selected class. Staff were present to assist participants with completing the questionnaire if they were having difficulties with reading the questions. Some participants chose not to participate because they did not have time or for other unknown reasons. Participants were not asked to provide identifying information, so the questionnaires were completed anonymously.

Demographic covariates included age, sex, Hispanic/Latino, language, insurance status, employment status, and self-reported health. Most participants were female (91.4%) and over 40 years (*n* = 216, 71.5%). The average age of participants was 48.2 years (SD = 13.9) with an age range of 18 to 86. There was roughly equal representation across employment status, preferred language, year of participation, and insurance status (Table 1).

### 2.3. Measures

The Godin-Shephard Leisure-Time Exercise Questionnaire (LTEQ) instrument was used to measure the intensity, frequency, and was modified to also measure duration of intentional physical activity [32,33]. The modified LTEQ contains three main questions regarding strenuous, moderate, and mild exercise behaviors within a 7-day period. The responses to these items determine the intensity of physical activity. Each question provides examples of activities related to the specified intensity and asks for both the average number of minutes exercised and how many times each week the participant exercises. Minutes and times to participate in exercise were used as estimates for time completing physical activity. Weekly frequency of moderate or strenuous physical activity was multiplied by the minutes spent on each moderate or strenuous activity to calculate each participant’s weekly metabolic equivalent adjusted minutes (MET minutes). The metric for being “physically active” was quantified as meeting the U.S. physical activity recommendations of ≥600 MET minutes weekly [34].

The Mental Health Continuum Short Form (MHC-SF) was included in the questionnaire and is a validated and reliable instrument for assessing emotional, psychological, and social well-being in English- and Spanish-speaking adults [35,36,37]. The MHC-SF contains 14 questions in which respondents report the frequency of positive mental health symptoms of emotional, social, and psychological well-being over the course of two weeks prior to completing the questionnaire. All questions were answered using a six-point Likert scale ranging from Never to Every Day. This instrument measured participant well-being and the total score was categorized into Languishing, Moderately Mentally Healthy, or Flourishing categories [36]. Higher scores indicate higher mental well-being (range of the total score was 0 to 70). Given the low sample size (*n* = 6) for languishing, it was combined with the moderate category.

### 2.4. Data Analysis

Simple descriptive statistics were created for all covariates, with standard deviations for continuous variables and counts and percentages for categorical variables. Chi-Square values were used to measure the association between mental well-being and all exercise covariates. Due to unexpected cell counts less than 5 for physical activity and frequency of class attendance, these variables were collapsed into binary variables. The Chi-Square test was used to determine whether an association existed between mental well-being and frequency of class attendance or the amount of self-reported MET minutes of activity. Logistic regression was used to explore these associations for all covariates. Unadjusted ORs (95% CI) models explored how each covariate individually is associated with mental well-being. All covariates with *p*-values < 0.25 were used to create a final adjusted model. Since participation in exercise classes was the exposure of interest, we evaluated all two-way interactions between exercise classes (MET minute classifications and number of classes attended) and all other covariates using an alpha of 0.01 to account for multiple testing. Additionally, classes were primarily populated by women; therefore, the over-representation of women is expected. During the analysis, gender was not associated with the outcome (mental health) individually or with an interaction with the exercise classes. SAS 9.4 was used to perform all analyses. An alpha of 0.05 was used to determine significance. Associations with *p*-values < 0.10 will be discussed along with effect sizes for hypothesis generation [38,39].

## 3. Results

Most participants in the study reported meeting the Federal Guidelines for weekly physical activity [40] using the self-reported MET minutes of moderate and strenuous intensity. Additionally, most participants self-reported a high frequency of positive mental well-being and were categorized as flourishing (75.2%) (Table 2).

### 3.1. Chi-Square Test

Using a significance level (alpha) equal to 0.05, no statistically significant associations between frequency of exercise class attendance and meeting physical activity guidelines with mental well-being were observed (Table 3).

### 3.2. Logistic Regression

Without adjustment, only age was statistically associated with mental well-being. Participants who were aged 60 years or older had 140% higher odds of reporting flourishing mental well-being relative to those who were less than 60 years old (Table 4). No other covariates were found to be associated with mental well-being among Hispanic participants. We further explored the association between covariates and mental well-being in models adjusted for age, employment status, self-reported health, frequency of class attendance, and meeting physical activity guidelines (*p*-values < 0.25). We excluded gender from adjusted models because gender was not associated with mental well-being and its addition to adjusted models did not change measures of association.

After adjustment, physical activity level was associated with mental well-being in Model 1 where all activity levels are considered: mild, moderate, and strenuous (Table 5). Those who engaged in moderate and strenuous self-reported physical activity self-reported 130% higher odds (OR = 2.30, 95% CI: 1.03, 5.12; *p*-value = 0.0422) of mental well-being adjusted for participants’ age, gender, and self-reported health. In adjusted Model 2, where only moderate and strenuous activity in self-reported physical activity level were considered, the association between mental well-being and intensity of physical activity was attenuated (OR = 1.89, 95% CI: 0.92, 3.9; *p*-value = 0.0848).

## 4. Discussion

This study provides evidence that meeting guidelines based on physical activity intensity and frequency is associated with mental well-being among a Hispanic population attending exercise classes. We found that meeting guidelines using moderate and strenuous physical activity as measured by the MHC-SF instrument was associated with 89% increased odds of flourishing mental well-being among low-income Hispanics participating in exercise classes. A study conducted with older Hispanic adults noted that physical activity was associated with positive emotions [41]. In general, exercise has been shown to improve mental health [42]. A study found that the optimal range of physical activity required to associate it with better mental health was between 2.5 to 7.5 h of physical activity a week [43]. Our study provides cross-sectional evidence that aligns with the current recommended physical activity guidelines [34].

Additionally, our study indicates that in older Hispanic populations, the consideration of mild physical activity along with moderate and strenuous physical activity is an important component to consider when studying the association between physical activity and flourishing mental health. Specifically, we observed that individuals who engaged in mild, moderate, and strenuous activity had 130% higher odds of positive mental well-being. This is an important finding and provides evidence that even engaging in mild activity, such as light 30-min walking, is associated with participant’s overall well-being.

The finding that engaging in mild, or light-intensity, physical activity to meet activity guidelines was associated with a flourishing mental health categorization strengthened the association between meeting activity guidelines and positive mental health among the same population of Hispanics participating in exercise classes. This might be especially important for participants who are new to exercise, those with limited time, or those who face other common obstacles to being able to perform moderate to high intensity exercise. A previous study also supported that mild physical activity was the optimal intensity for women to report positive mental health, but this same study found that the optimal physical activity intensity for men for positive mental health was high intensity [21]. Interestingly, studies have found that light-intensity physical activity is also associated with many physical health benefits as well as meaningful improvements in mental health related to depressive symptoms in both the general adult and older adult populations [44,45,46]. Our study supports this evolving evidence that mild, moderate, and strenuous is associated with positive mental health and our findings suggest that attendance in group exercise classes in low-income communities is associated with positive mental health.

Regarding the amount of time spent participating in physical activity, as shown in Table 2, participants who reported attending exercise classes one to multiple times a week were more likely to belong to the flourishing mental health category (82.7%) as opposed to those who reported attending exercise classes none to several times a month (17.3%). Other studies suggested that higher participation or exercise class attendance may be explained by the social support aspect of group exercise classes [12].

A study conducted on Scottish adults indicated some estimates of time and intensity as it is associated with improved mental health. This study found that a minimum of 20 min of physical activity a week was associated with observed mental health benefits. The higher the participation and/or intensity the greater the mental health benefits [47]. The average length of a TSSC exercise class is 60 min, so participants attending one or more times a week easily achieved more than the 20 min observed by Hamer et al. [47]. In fact, attending two to three free TSSC exercise classes per week can help participants meet U.S. physical activity guidelines [34].

Our findings suggest that frequent attendance at free TSSC exercise classes may be associated with self-reported flourishing mental health. Although neither the unadjusted nor adjusted associations were significant, the *p*-values were <0.10. Due to the nature of how the questions were asked, 121 observations were not included in the study due to issues with responses dealing with exercise frequency and intensity. With the inclusion of these responses, if this trend towards association continued, it is likely that the relationship would meet the traditional significance cutoff. This larger sample size would have made comparisons between the subgroups more adequately powered to detect the associations. Due to these response issues, future iterations of the survey should incorporate more thoroughly describing the questions and be constrained to not allow for incorrect responses. Future studies should also examine the extent of the effect of social support as a factor influencing mental health through group exercise classes. Follow-up studies could also explore the effects of providing a safe infrastructure and active transportation to create opportunities for moderate and strenuous physical activity as well as activating public spaces for active transportation (i.e., walking and hiking) to ensure mild physical activity effects on mental health well-being.

### Limitations

There are several limitations to consider in this study. Firstly, this cross-sectional study did not include a no-treatment control group. Therefore, we are unable to draw conclusions about flourishing and languishing mental health status among a group of Hispanic individuals not participating in exercise classes. Additionally, because these are cross-sectional data, it is not possible to determine causality between exercise class attendance and positive mental health. We are also not able to determine if participants who self-reported flourishing mental health were more likely to attend the exercise classes or whether attendance contributes to a flourishing mental health status. Given the concatenation of languishing and moderate groups we could also not distinguish effectiveness of the classes to affect changes between these groupings. Another limitation is that participants self-reported their physical activity levels and positive mental health. It is possible that both variables could be reported more favorably. The sample was largely female as all classes are predominantly attended by women, with only 8.6% male participants overall. Therefore, the associations reported are more robustly sound for Hispanic women. Engaging men in the TSSC exercise offerings is an ongoing challenge that is being addressed by providing various exercise options, including those centered around strength training and boot camps. Lastly, because identifying information was not collected, we were unable to determine whether a participant submitted multiple questionnaires either by attending two of the selected classes in one year, or by completing a questionnaire both years. As such, all data points may not be unique and some of the data may be duplicated.

## 5. Conclusions

Using the LTEQ and MHC-SF, this study supported the hypothesis, showing that all levels of physical activity were found to be associated with self-reported flourishing mental health among participants in the free, culturally relevant, exercise class offerings among low-income Mexican American Hispanics participating in the Tu Salud ¡Si Cuenta! (TSSC) program. Future studies may compare the effect of exercise class participants to a sedentary group, all of whom receive TSSC nutrition education, to further evaluate the effect of TSSC exercise class participation on self-reported mental health outcomes. Furthermore, the results lend themselves to further study on the effect of group exercise versus individual exercise on mental health. The study’s findings address gaps in the literature regarding the impact of physical activity on Mexican Americans’ mental well-being, contribute to existing research about the effect of physical activity on general mental health, and can provide insight into the planning and development of community programming tailored to low-income populations.

## Figures and Tables

**Table 1:** Demographic characteristics for Hispanic participants attending free community exercise classes, 2018–2019 (*n* = 302).

Variable	Class	*n* (%)
Age Category	18–29	28 (9.3)
	30–39	58 (19.2)
	40–49	77 (25.5)
	50–59	74 (24.5)
	60+	65 (21.5)
Sex	M	26 (8.6)
	F	276 (91.4)
Preferred Language	English	161 (53.3)
	Spanish	141 (46.7)
Year	2018	149 (49.3)
	2019	153 (50.7)
Insurance Status	No	141 (48.0)
	Yes	153 (52.0)
Employment Status	Unemployed (all reasons)	151 (52.8)
	Employed	135 (47.2)
Self-Reported Health Status	Poor/Fair/Not Sure	83 (28.2)
	Good	129 (43.7)
	Very Good	50 (17.0)
	Excellent	33 (11.2)

**Table 2:** Levels of physical activity, frequency of class attendance, and mental well-being for Hispanic participants attending free community exercise classes, 2018–2019 (*n* = 302).

Variable	Class	*n* (%)
Physical Activity Intensity Level 1 (mild, moderate, strenuous)	Sedentary/Low (did not meet recommendations)	42 (13.9)
	Mild/Mod/Strenuous (met recommendations)	260 (86.1)
Physical Activity Intensity Level 2 (moderate, strenuous)	Sedentary/Low (did not meet recommendations)	61 (20.2)
	Mod/Strenuous (met recommendations)	241 (79.8)
Time in Class Attendance	<1 to several times a month or my first week	59 (19.9)
	At least once weekly to multiple times per week	238 (80.1)
MHC-SF Diagnosis of Mental Well-Being	Languishing/Moderate	75 (24.8)
	Flourishing	227 (75.2)

**Table 3:** Chi-Square tests for the association between self-reported mental well-being and frequency of class attendance and levels of physical activity among Hispanic participants.

	Mental Well-Being		
	Languishing and Moderate *n* (%)	Flourishing *n* (%)	χ^2^ *p*-Value
Frequency Attending Community Exercise Class			0.0532
None to several times a month or first time	20 (27.8)	39 (17.3)	
One to multiple times a week	52 (72.2)	186 (82.7)	
Mild, Moderate, and Strenuous Intensity of Physical Activity (MET-adjusted minutes)			0.0786
Sedentary to Low (did not meet recommendations)	15 (20.0)	27 (11.9)	
Mild, Moderate, Strenuous (met recommendations)	60 (80.0)	200 (88.1)	
Moderate and Strenuous Intensity of Physical Activity (MET-adjusted minutes)			0.2014
Sedentary to Low (did not meet recommendations)	19 (25.3)	42 (18.5)	
Moderate, Strenuous (met recommendations)	56 (74.7)	185 (81.5)	

**Table 4:** Unadjusted binary logistic regression predicting “flourishing” mental well-being among Hispanic participants—ORs (95% CI and *p*-value).

Variable	Class	OR (95% CI)	*p*-Value
Age Category	60+ vs. <60	2.40 (1.12, 5.13)	0.0237
Sex	Male vs. Female	0.59 (0.25, 1.39)	0.2310
Insurance	No vs. Yes	0.75 (0.44, 1.27)	0.2817
Employment Status	Currently Unemployed vs. Employed	0.68 (0.4, 1.18)	0.1738
Self-Reported Health	Good vs. Poor/Fair/Not Sure	1.51 (0.83, 2.76)	0.1440
	Very Good vs. Poor/Fair/Not Sure	2.72 (1.13, 6.58)	0.4798
	Excellent vs. Poor/Fair/Not Sure	5.18 (1.45, 18.47)	0.0612
How Often Do You Attend Class	None to Several Times a Month vs.One to Several Times a Week	1.83 (0.99, 3.41)	0.0554
Physical Activity Level	Moderate and Strenuous (met recommendations) vs. Sedentary and Low (did not meet recommendations)	1.85 (0.93, 3.71)	0.0818
Physical Activity Level †	Moderate and Strenuous (met recommendations) vs. Sedentary and Low (did not meet recommendations)	1.50 (0.81, 2.78)	0.2031

† moderate, strenuous.

**Table 5:** Adjusted binary logistic regression results of association with mental well-being among Hispanic participants—ORs (95% CI and *p*-value).

Variable	Class	Physical Activity Level			
		Model 1 Mild, Moderate, Strenuous		Model 2 Moderate, Strenuous	
		OR (95% CI)	*p*-Value	OR (95% CI)	*p*-Value
Age Category	60+ vs. <60	2.21 (0.97, 5.06)	0.0596	2.22 (0.97, 5.1)	0.0595
Self-Reported Health	Good vs. Poor/Fair/Not Sure	1.72 (0.9, 3.29)	0.5062	1.7 (0.89, 3.26)	0.4692
	Very Good vs. Poor/Fair/Not Sure	2.28 (0.91, 5.69)	0.7382	2.28 (0.91, 5.68)	0.7520
	Excellent vs. Poor/Fair/Not Sure	4.36 (1.19, 15.93)	0.1098	4.5 (1.23, 16.41)	0.0974
How Often Do You Attend Class	At least once weekly to multiple times per week vs. <1 to several times a month or my first week	1.75 (0.89, 3.44)	0.1075	1.79 (0.91, 3.52)	0.0904
Physical Activity Level	Moderate and Strenuous (met guidelines) vs. Sedentary and Low (did not meet guidelines)	2.30 (1.03, 5.12)	0.0422	1.89 (0.92, 3.9)	0.0848

## Data Availability

The data presented in this study are available on request from the corresponding author.

## References

[1] Reiner M., Niermann C., Jekauc D., Woll A. (2013). Long-term health benefits of physical activity—A systematic review of longitudinal studies. BMC Public Health.

[2] Cleven L., Krell-Roesch J., Nigg C.R., Woll A. (2020). The association between physical activity with incident obesity, coronary heart disease, diabetes and hypertension in adults: A systematic review of longitudinal studies published after 2012. BMC Public Health.

[3] Amesty S.C. (2003). Barriers to Physical Activity in the Hispanic Community. J. Public Health Policy.

[4] Bautista L., Reininger B., Gay J.L., Barroso C.S., McCormick J.B. (2011). Perceived barriers to exercise in Hispanic adults by level of activity. J. Phys. Act. Health.

[5] Reininger B., Wang J., Cron S., Fisher-Hoch S.P. (2012). Preventive Health Behaviors among Hispanics: Comparing A US-Mexico Border Cohort and National Sample. Int. J. Exerc. Sci. Conf. Proc..

[6] Heredia N.I., Lee M., Mitchell-Bennett L., Reininger B.M. (2017). Tu Salud ¡Sí Cuenta! Your Health Matters! A Community-wide Campaign in a Hispanic Border Community in Texas. J. Nutr. Educ. Behav..

[7] Hovell M.F., Mulvihill M.M., Buono M.J., Liles S., Schade D.H., Washington T.A., Manzano R., Sallis J.F. (2008). Culturally tailored aerobic exercise intervention for low-income Latinas. Am. J. Health Promot..

[8] Vincent D. (2009). Culturally tailored education to promote lifestyle change in Mexican Americans with type 2 diabetes. J. Am. Acad. Nurse Pract..

[9] Ickes M.J., Sharma M. (2012). A systematic review of physical activity interventions in Hispanic adults. J. Environ. Public Health.

[10] Heredia N.I., Lee M., Reininger B.M. (2017). Exposure to a community-wide campaign is associated with physical activity and sedentary behavior among Hispanic adults on the Texas-Mexico border. BMC Public Health.

[11] Kouvonen A., De Vogli R., Stafford M., Shipley M.J., Marmot M.G., Cox T., Vahtera J., Vaananen A., Heponiemi T., Singh-Manoux A. (2012). Social support and the likelihood of maintaining and improving levels of physical activity: The Whitehall II Study. Eur. J. Public Health.

[12] Lindsay Smith G., Banting L., Eime R., O’Sullivan G., van Uffelen J.G.Z. (2017). The association between social support and physical activity in older adults: A systematic review. Int. J. Behav. Nutr. Phys. Act..

[13] Graupensperger S., Gottschall J.S., Benson A.J., Eys M., Hastings B., Evans M.B. (2019). Perceptions of groupness during fitness classes positively predict recalled perceptions of exertion, enjoyment, and affective valence: An intensive longitudinal investigation. Sport Exerc. Perform. Psychol..

[14] Heinrich K., Kurtz B., Patterson M., Crawford D., Barry A. (2022). Incorporating a Sense of Community in a Group Exercise Intervention Facilitates Adherence. Health Behav. Res..

[15] Firestone M.J., Yi S.S., Bartley K.F., Eisenhower D.L. (2015). Perceptions and the role of group exercise among New York City adults, 2010–2011: An examination of interpersonal factors and leisure-time physical activity. Prev. Med..

[16] Stevens M., Rees T., Polman R. (2019). Social identification, exercise participation, and positive exercise experiences: Evidence from parkrun. J. Sports Sci..

[17] Lee B., Howard E.P. (2019). Physical Activity and Positive Psychological Well-Being Attributes Among U.S. Latino Older Adults. J. Gerontol. Nurs..

[18] Schuch F.B., Vancampfort D. (2021). Physical activity, exercise, and mental disorders: It is time to move on. Trends Psychiatry Psychother..

[19] Gomes M., Figueiredo D., Teixeira L., Poveda V., Paúl C., Santos-Silva A., Costa E. (2017). Physical inactivity among older adults across Europe based on the SHARE database. Age Ageing.

[20] Tamminen N., Reinikainen J., Appelqvist-Schmidlechner K., Borodulin K., Mäki-Opas T., Solin P. (2020). Associations of physical activity with positive mental health: A population-based study. Ment. Health Phys. Act..

[21] Asztalos M., De Bourdeaudhuij I., Cardon G. (2010). The relationship between physical activity and mental health varies across activity intensity levels and dimensions of mental health among women and men. Public Health Nutr..

[22] McFadden T., Fortier M., Sweet S.N., Tomasone J.R. (2021). Physical activity participation and mental health profiles in Canadian medical students: Latent profile analysis using continuous latent profile indicators. Psychol. Health Med..

[23] Felez-Nobrega M., Bort-Roig J., Ma R., Romano E., Faires M., Stubbs B., Stamatakis E., Olaya B., Haro J.M., Smith L. (2021). Light-intensity physical activity and mental ill health: A systematic review of observational studies in the general population. Int. J. Behav. Nutr. Phys. Act..

[24] Hernandez R., Andrade F.C.D., Piedra L.M., Tabb K.M., Xu S., Sarkisian C. (2019). The impact of exercise on depressive symptoms in older Hispanic/Latino adults: Results from the ‘¡Caminemos!’ study. Aging Ment. Health.

[25] Walsh J.L., Senn T.E., Carey M.P. (2013). Longitudinal associations between health behaviors and mental health in low-income adults. Transl. Behav. Med..

[26] US Census Bureau (2020). DEC Redistricting Data (PL 94-171). https://www.census.gov/programs-surveys/decennial-census/about/rdo/summary-files.html.

[27] US Census Bureau (2020). American Community Survey 5-Year Estimates Subject Tables. https://www.census.gov/acs/www/data/data-tables-and-tools/subject-tables/.

[28] Fisher-Hoch S.P., Rentfro A.R., Salinas J.J., Perez A., Brown H.S., Reininger B.M., Restrepo B.I., Wilson J.G., Hossain M.M., Rahbar M.H. (2010). Socioeconomic status and prevalence of obesity and diabetes in a Mexican American community, Cameron County, Texas, 2004–2007. Prev. Chronic Dis..

[29] Alzoubi A., Abunaser R., Khassawneh A., Alfaqih M., Khasawneh A., Abdo N. (2018). The Bidirectional Relationship between Diabetes and Depression: A Literature Review. Korean J. Fam. Med..

[30] Task Force on Community Preventive Services (2002). Recommendations to increase physical activity in communities. Am. J. Prev. Med..

[31] Yeh P.G., Reininger B.M., Mitchell-Bennett L.A., Lee M., Xu T., Davé A.C., Park S.K., Ochoa-Del Toro A.G. (2022). Evaluating the Dissemination and Implementation of a Community Health Worker-Based Community Wide Campaign to Improve Fruit and Vegetable Intake and Physical Activity among Latinos along the U.S.-Mexico Border. Int. J. Environ. Res. Public Health.

[32] Godin G., Shephard R.J. (1985). A simple method to assess exercise behavior in the community. Can. J. Appl. Sport Sci..

[33] Vidoni M.L., Lee M., Mitchell-Bennett L., Reininger B.M. (2019). Home Visit Intervention Promotes Lifestyle Changes: Results of an RCT in Mexican Americans. Am. J. Prev. Med..

[34] Piercy K.L., Troiano R.P., Ballard R.M., Carlson S.A., Fulton J.E., Galuska D.A., George S.M., Olson R.D. (2018). The Physical Activity Guidelines for Americans. JAMA.

[35] Keyes C.L.M. (1998). Social Well-Being. Soc. Psychol. Q..

[36] Lamers S.M., Westerhof G.J., Bohlmeijer E.T., ten Klooster P.M., Keyes C.L. (2011). Evaluating the psychometric properties of the Mental Health Continuum-Short Form (MHC-SF). J. Clin. Psychol..

[37] Echeverría G., Torres M., Pedrals N., Padilla O., Rigotti A., Bitran M. (2017). Validation of a Spanish Version of the Mental Health Continuum-Short Form Questionnaire. Psicothema.

[38] Aguinis H., Vassar M., Wayant C. (2021). On reporting and interpreting statistical significance and p values in medical research. BMJ Evid.-Based Med..

[39] Di Leo G., Sardanelli F. (2020). Statistical significance: P value, 0.05 threshold, and applications to radiomics—Reasons for a conservative approach. Eur. Radiol. Exp..

[40] US Department of Health and Human Services (HHS) Physical Activity Guidelines for Americans: Be Active, Healthy, and Happy! 2008. www.health.gov/paguidelines.

[41] Rizvi S., Khan A.M. (2019). Physical Activity and Its Association with Depression in the Diabetic Hispanic Population. Cureus.

[42] Mikkelsen K., Stojanovska L., Polenakovic M., Bosevski M., Apostolopoulos V. (2017). Exercise and mental health. Maturitas.

[43] Kim Y.S., Park Y.S., Allegrante J.P., Marks R., Ok H., Ok Cho K., Garber C.E. (2012). Relationship between physical activity and general mental health. Prev. Med..

[44] Füzéki E., Engeroff T., Banzer W. (2017). Health Benefits of Light-Intensity Physical Activity: A Systematic Review of Accelerometer Data of the National Health and Nutrition Examination Survey (NHANES). Sports Med..

[45] Loprinzi P.D. (2017). Light-Intensity Physical Activity and All-Cause Mortality. Am. J. Health Promot..

[46] Ku P.W., Steptoe A., Liao Y., Sun W.J., Chen L.J. (2018). Prospective relationship between objectively measured light physical activity and depressive symptoms in later life. Int. J. Geriatr. Psychiatry.

[47] Hamer M., Stamatakis E., Steptoe A. (2009). Dose-response relationship between physical activity and mental health: The Scottish Health Survey. Br. J. Sports Med..

